# TGFβ1‐induced beta‐site APP‐cleaving enzyme 2 upregulation promotes tumorigenesis through the NF‐κB signalling pathway in human gliomas

**DOI:** 10.1002/1878-0261.12623

**Published:** 2020-01-07

**Authors:** Huizhi Wang, Zihang Chen, Shaobo Wang, Xiao Gao, Mingyu Qian, Wei Qiu, Zongpu Zhang, Shouji Zhang, Yanhua Qi, Xiaopeng Sun, Hao Xue, Xing Guo, Rongrong Zhao, Gang Li

**Affiliations:** ^1^ Department of Neurosurgery Qilu Hospital of Shandong University Jinan Shandong Province China; ^2^ Institute of Brain and Brain‐Inspired Science Shandong University Jinan Shandong Province China; ^3^ Shandong Key Laboratory of Brain Function Remodeling Shandong University Jinan Shandong Province China; ^4^ Department of Neurosurgery Dezhou People's Hospital Dezhou Shandong Province China

**Keywords:** BACE2, cell cycle, EMT, glioma, NF‐κB signalling, TGFβ signalling

## Abstract

Gliomas are the most common primary malignant tumours of the central nervous system, and new molecular biomarkers are urgently needed for diagnosis and targeted therapy. Here, we report that increased beta‐site APP‐cleaving enzyme 2 (BACE2) expression is associated with increases in the grade of human glioma, the incidence of the mesenchymal molecular glioblastoma multiforme subtype and the likelihood of poor prognoses for patients. BACE2 knockdown suppressed cell invasion, cell migration and tumour growth both *in vitro* and *in vivo*, while BACE2 overexpression promoted the mesenchymal transition and cell proliferation. Furthermore, TGFβ1 stimulated BACE2 expression through Smad‐dependent signalling, which modulated TNF‐α‐induced NF‐κB activity through the PP1A/IKK pathway to promote tumorigenesis in both U87MG and U251 cells. Our study indicated that BACE2 plays a significant role in glioma development. Therefore, BACE2 is a potential therapeutic target for human gliomas due to its function and ability to be regulated.

## Graphical abstract

This study reports TGFβ1 stimulates beta‐site APP‐cleaving enzyme 2 (BACE2) expression through Smad‐dependent signalling, which modulates TNF‐α‐induced NF‐κB activity through the PP1A/IKK pathway to promote tumorigenesis in glioma cells. BACE2 knockdown suppressed cell invasion, cell migration and tumour growth both *in vitro* and *in vivo*, while BACE2 overexpression promoted the mesenchymal transition and cell proliferation.

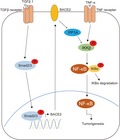

**Abbreviations**
AD: Alzheimer's disease
ATRX: alpha‐thalassaemia/mental retardation syndrome X‐linked
BACE2: beta‐site APP‐cleaving enzyme 2
CCK‐8: Cell Counting Kit‐8
CGGA: The Chinese Glioma Genome Atlas
EMT: epithelial–mesenchymal transition
GBM: glioblastoma multiforme
GO: Gene Ontology
GSEA: gene set enrichment analysis
IDH1: isocitrate dehydrogenase 1
IHC: immunohistochemistry
IP: immunoprecipitation
KEGG: Kyoto Encyclopaedia of Genes and Genomes
LGGs: low‐grade gliomas
MGMT: methylation of *O*‐methylguanine‐DNA methyltransferase
PI: propidium iodide
RT‐qPCR: real‐time qPCR
siRNA: small‐interfering RNA
TCGA: The Cancer Genome Atlas
TERT: telomerase reverse transcriptase
WHO: World Health Organization

## 1 Introduction

Human gliomas are reported to be the most common type of primary intracranial tumour, accounting for ~ 80% of all malignant brain tumours. According to the World Health Organization (WHO), gliomas can be classified into four grades (I–IV) according to histological features, with grade IV tumours or glioblastoma multiforme (GBM) being associated with the worst prognosis, namely a median survival time of just 12–15 months and a 5‐year survival rate < 3% after initial diagnosis (Torre *et al.*, 2015; Wen and Kesari, 2008).

Beta‐site APP‐cleaving enzyme 2 (BACE2) is an integral membrane glycoprotein belonging to the peptidase A1 family and has a molecular weight of 56 kDa. Prior studies have noted the importance of BACE2 in Alzheimer's disease (AD) and type 2 diabetes (Esler and Wolfe, 2001; Esterhazy *et al.*, 2011). In addition, previous studies have shown that BACE2 is upregulated in primary breast cancer and colorectal cancer as well as in colorectal adenomas (Shao *et al.*, 2018; Tsuji *et al.*, 2004), thus indicating the key role for BACE2 in malignant tumour progression. However, few reports on BACE2 expression patterns, the clinical significance of BACE2 in human gliomas and the underlying mechanisms of BACE2 function were found in the literature.

In the present study, we used publicly datasets to investigate the relationship between BACE2 expression levels and tumour grade or the clinicopathological and molecular features of gliomas. We further explored the function of BACE2 both *in vitro* and *in vivo*. Moreover, we investigated the potential molecular mechanisms of its function. Taken together, our findings indicate that BACE2 might be a novel prognostic biomarker and a potential therapeutic target for human gliomas.

## 2 Materials and methods

### 2.1 Cell line culture

We obtained U87MG and U251 human GBM cells from the Culture Collection of the Chinese Academy of Sciences. U87MG and U251 cells were cultured in Dulbecco's modified Eagle's medium (Thermo Fisher Scientific, Grand Island, NY, USA) supplemented with 10% FBS (Thermo Fisher Scientific) (Sun *et al.*, 2019).

### 2.2 Tissue preparation

Clinical samples including six WHO II tissues, seven WHO Ⅲ tissues and 12 GBM tissues were collected from patients who underwent glioma resection surgery in the Department of Neurosurgery of Qilu Hospital at Shandong University. Normal brain tissue samples (*n* = 4) were collected from patients who underwent craniocerebral decompression treatment for brain trauma. Subsequently, the tissues were embedded in paraffin for immunohistochemistry analysis. For real‐time qPCR (RT‐qPCR) analysis, samples including 21 low‐grade glioma (LGG) tissues, 13 GBM tissues and 6 normal brain tissues were immediately stored in liquid nitrogen after surgery and kept until RNA extraction. The use of tumour tissues for study was approved by the Institutional Review Board of Qilu Hospital in compliance with the Helsinki Declaration.

### 2.3 Immunohistochemistry

Sections were obtained from paraffin‐embedded clinical sample tissues and xenograft models. Then, tumour sections were examined with immunohistochemistry (IHC) as previously described (Xue *et al.*, 2016). IHC staining was performed with anti‐BACE2, anti‐N‐cadherin and anti‐Ki‐67.

### 2.4 RNA interference

Small‐interfering RNA (siRNA) targeting BACE2 was synthesized. On the basis of the manufacturer's protocol, cells were transfected with siRNA as previously described (Han *et al.*, 2017). The efficient siRNA sequences (*n* = 2) were as follows: si‐BACE2#1: 5′ACAGAGAGGUCUAGCACAUTT′ and si‐BACE2#2: 5′GGGAUUAAAUGGAAUGGAATT3′. The glioma cells used for functional assays *in vitro* were transfected with si‐BACE2#1.

### 2.5 Lentivirus transfection

Stable overexpression of BACE2 and the control genes in cells was achieved by transfection with lentivirus synthesized by GeneChem (Shanghai, China). The cells were transfected with lentivirus as previously described (Han *et al.*, 2017).

### 2.6 Real‐time qPCR

RNA was extracted from cells or tissues derived from glioma patients with TRIzol. RT‐qPCR was performed as previously described (Xue *et al.*, 2016). Primers are listed in Table S1.

### 2.7 Western blot analysis

Total proteins were obtained from harvest cells in RIPA lysis buffer containing 50 mm Tris (pH 7.4), 150 mm NaCl, 1% sodium deoxycholate, 0.1% SDS, 20 mm EDTA, 1 mm NaF and 1% Triton X‐100 with 2 mm sodium pyrophosphate as previously described (Xue *et al.*, 2016). The following antibodies were used: BACE2 (ab5670), N‐cadherin (#13116), E‐cadherin (#3195), β‐catenin (#8480), MMP2 (#40994), Vimentin (#5741), Snail (#3879), Twist (#46702), CDK2 (#2546), CDK4 (#12790), Cyclin D1 (#2978), c‐Myc (#13987), p21 (#2947), p27 (#3686), p65 (#8242), p‐p65 (#3033), Smad2 (#5339), p‐Smad2 (#18338), PP1A (#2582), p‐PP1A (#2581), IKKβ (#8943), p‐IKKβ (#2694), IKBα (#11930), p‐IKBα (#2859) and GAPDH (#5174).

### 2.8 Immunoprecipitation

The protein of glioma cells was obtained with a Pierce™ Classic Magnetic immunoprecipitation (IP)/Co‐IP Kit (Thermo Fisher). Then, western blotting was performed as indicated.

### 2.9 3D tumour spheroid invasion assay

The 3D tumour spheroid invasion assay was performed as previously described (Han *et al.*, 2017). The cells with spheroids were photographed every 24 h using Nikon microscopy and quantitatively analysed by ImageJ (NIH, Bethesda, MD, USA).

### 2.10 Transwell, wound‐healing and cell proliferation assays

Migration and invasion assays were performed as previously described (Qian *et al.*, 2019). Five random fields from each well were photographed, and the cell numbers were counted by imagej. 1.2 × 10^6^ cells were seeded in six‐well plates and incubated for 2 days after transfection. Then, the wound‐healing assay was performed as previously described (Sun *et al.*, 2019). The line in the cells was photographed at 0 and 48 h by a Nikon microscope. According to the manufacturer's protocol, the ability of cell proliferation of glioma cells was determined with EdU assay kit (RiboBio, Guangzhou, China) and Cell Counting Kit‐8 (CCK‐8; Dojindo, Kumamoto, Japan).

### 2.11 Immunofluorescence

The transfected cells were seeded on circular slides in a 12‐well plate for 24 h. The slides were stained with FITC‐labelled phalloidin, and the cell nuclei were stained with DAPI as previously described (Han *et al.*, 2017). The slides were photographed with a Nikon inverted fluorescence microscope.

### 2.12 Flow cytometry

According to the manufacturer's instructions, transfected cells were stained with propidium iodide (PI). Flow cytometry was performed using BD Accuri C6 flow cytometer (BD Biosciences, San Diego, CA, USA) and quantitatively analysed by modfit lt 4.0 (Verity Software House Inc, Topsham, ME, USA).

### 2.13 Luciferase reporter assay

Glioma cells transfected with NF‐κB reporter vector p‐NF‐κB‐luc (Beyotime, Haimen, China) were transfected with siBACE2, siPP1A or negative control siRNA. According to the manufacturer's protocol, the Dual Luciferase Reporter kit (Beyotime) was used to determine luciferase activity.

### 2.14 *In vivo* imaging of implants in nude mice

shBACE2 cells and the corresponding control luciferase‐labelled U87MG cells for use *in vivo* were transfected with lentivirus synthesized by GeneChem. Cells (1 × 10^6^) were implanted into the brains of nude mice with solid brain positioners. Mice were imaged *in vivo* with a bioluminescence imaging system, namely IVIS Lumina Series III (PerkinElmer, Waltham, MA, USA), 7 and 14 days after implantation. Luciferin was injected intraperitoneally after the nude mice were subjected to gas anaesthesia. Five minutes later, the tumour volume was measured and quantified. After extraction, tumour tissues were embedded in paraffin and incubated with BACE2, N‐cadherin and Ki‐67 antibodies.

### 2.15 Public datasets

Transcriptome data of glioma samples and the corresponding clinical information were obtained from The Cancer Genome Atlas Research Network (TCGA; *n* = 667; http://cancergenome.nih.gov). Quantile normalization was used to normalize mRNA expression [transcription fragments per million base pairs per thousand base fragments (FPKM)] data. mRNA microarray and matched clinical data of Chinese glioma patients were downloaded from samples in the Chinese Glioma Genome Atlas (CGGA; *n* = 325; http://www.cgga.org.cn), and mRNA expression data were normalized with *Z*‐Score. The http://www.ncbi.nlm.nih.gov/geo/query/acc.cgi?acc=GSE16011 (*n* = 284), http://www.ncbi.nlm.nih.gov/geo/query/acc.cgi?acc=GSE4290 (*n* = 180) and http://www.ncbi.nlm.nih.gov/geo/query/acc.cgi?acc=GSE4271 (*n* = 100) datasets were downloaded from the Gene Expression Omnibus database (https://www.ncbi.nlm.nih.gov/geo).

### 2.16 Gene Ontology (GO) and Kyoto Encyclopaedia of Genes and Genomes analysis

Correlation analysis for the transcriptome profile of BACE2 was performed in the TCGA database using the edgeR package with r version 3.5.1. The DAVID web tool (http://david.abcc.ncifcrf.gov/home.jsp) was used to analyse the positive and negative genes (|fold change| > 1.5; *P* < 0.01) associated with BACE2 expression to study biological processes and Kyoto Encyclopaedia of Genes and Genomes (KEGG) signalling pathways in gliomas (Han *et al.*, 2017). Gene set enrichment analysis (gsea) software (http://software.broadinstitute.org/) was used to analyse the association between BACE2 expression and the specific gene sets from the Molecular Signatures Database as previously described (Subramanian *et al.*, 2005).

### 2.17 Cox proportional hazards model

Univariate and multivariate analyses were conducted with the Cox proportional hazards model using the data from the TCGA database. For this study, five variables were included: age and sex (patient factors); WHO grade and IDH status (glioma characteristics); and BACE2 mRNA expression. WHO grade IV gliomas, which were categorized as WHO high‐grade gliomas, were compared with WHO grade II and WHO grade Ⅲ gliomas, which were categorized as WHO LGGs. A BACE2 mRNA expression level that exceeded the median value was defined as high BACE2 expression; conversely, a BACE2 mRNA expression level that fell below this value was defined as low BACE2 expression.

### 2.18 Transcriptome sequencing

U87MG cells were transfected with siBACE2#1 or negative control siRNA. Forty‐eight hours later, total RNA was isolated from control and BACE2‐knockdown U87MG cells, and stored at −80 °C for subsequent sequencing. Transcriptome sequencing was performed as previously described (Qian *et al.*, 2019). Enrichment analysis of BACE2‐related genes (|fold change| > 0.8; *P* < 0.05) in U87MG cells was performed as indicated.

### 2.19 Statistical analysis

spss 22.0 software (IBM, Armonk, NY, USA) was used for data analysis. graphpad prism 6 software (GraphPad Software Inc., La Jolla, CA, USA) was used for data visualization. Kaplan–Meier method, Student's *t*‐test, one‐way ANOVA, two‐tailed chi‐square test and Pearson's correlation coefficient were used for analysing statistical significance of all data. From the univariate analysis, statistically significant variables were selected for the subsequent multivariate Cox proportional hazards regression model analysis. All results are shown as the mean ± standard error. *P*‐values < 0.05 (two‐tailed) were considered significantly.

## 3 Results

### 3.1 Higher expression levels of BACE2 are associated with a higher grade of human glioma

**Figure 1:**
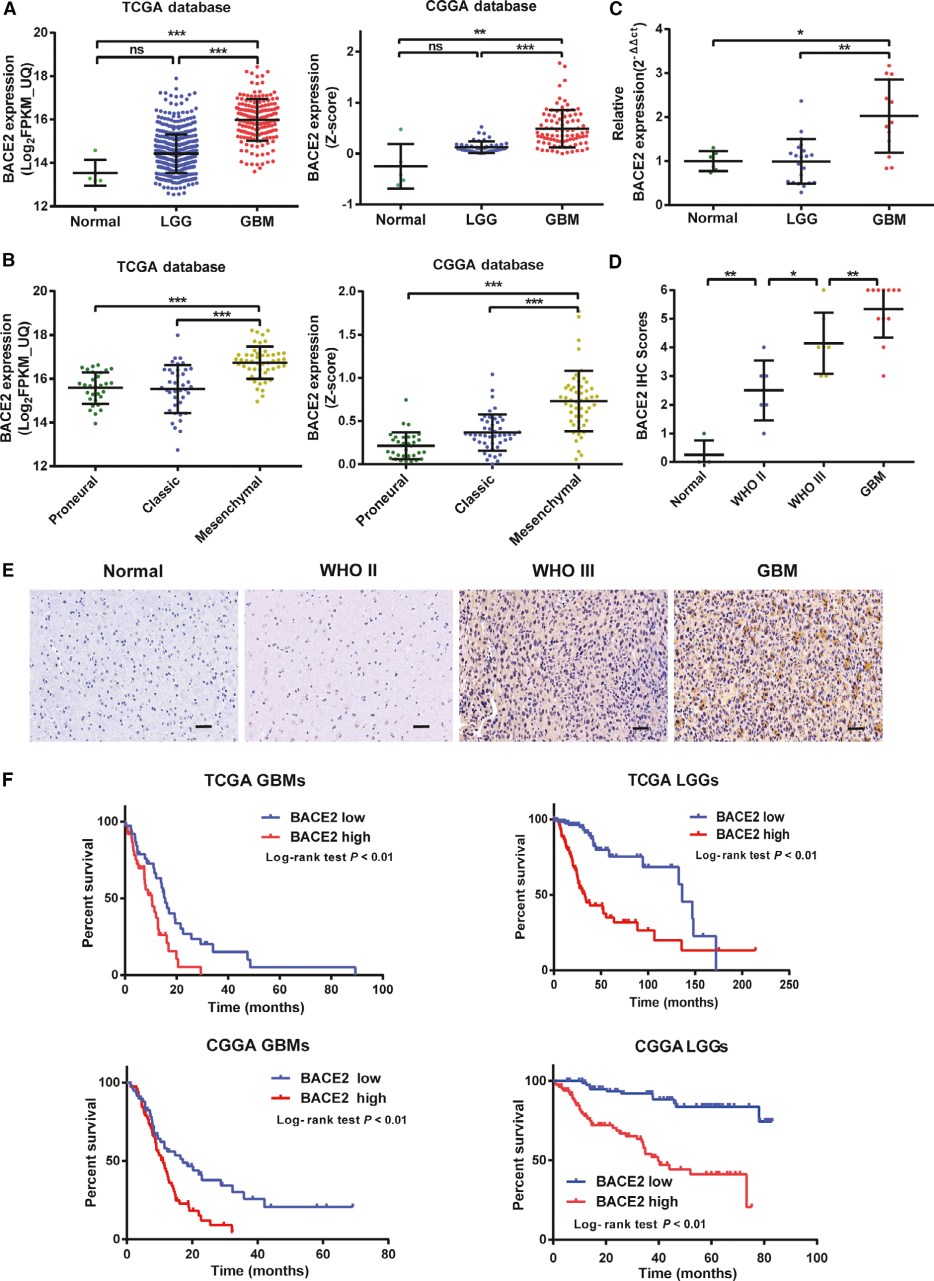
BACE2 expression is associated with tumour grade in gliomas. (A) Results of the quantification of BACE2 expression in glioma tissues with the TCGA and CGGA databases. (B) Results of the quantification of BACE2 expression in different subtypes of GBM cells with the TCGA and CGGA databases. (C) Real‐time PCR analysis of BACE2 expression in human glioma and normal brain tissue samples. GAPDH was used as a loading control. (D) Graphic representation of scoring performed on IHC for BACE2 in glioma tissues and normal brain tissues. (E) BACE2 protein levels were analysed by IHC in human glioma and normal brain tissue samples. Magnification: 400×. (F) The prognostic value of BACE2 expression in the LGG and GBM was analysed with the TCGA database (*n* = 667) and the CGGA database (*n* = 272). The cut‐off was the median BACE2 expression level. ***P < 0.001; **P < 0.01; *P < 0.05; ns, not significant.

To determine the role of BACE2 in the development of glioma, the expression of BACE2 was analysed in normal brain tissues, LGG tissues and GBM tissues. Compared to that in the LGGs and normal brain tissues, BACE2 mRNA expression was significantly upregulated in the GBM tissues in the TCGA and CGGA databases (Fig. 1A). However, there is no significant difference in BACE2 expression between the LGGs and normal brain tissues in either database. We verified these findings in three other published datasets, namely http://www.ncbi.nlm.nih.gov/geo/query/acc.cgi?acc=GSE16011, http://www.ncbi.nlm.nih.gov/geo/query/acc.cgi?acc=GSE4290 and http://www.ncbi.nlm.nih.gov/geo/query/acc.cgi?acc=GSE4271. BACE2 expression was also increased in the GBM tissues relative to the LGG and normal brain tissues in these datasets (Fig. S1A). BACE2 expression of the normal brain tissues and LGGs in the http://www.ncbi.nlm.nih.gov/geo/query/acc.cgi?acc=GSE16011 and http://www.ncbi.nlm.nih.gov/geo/query/acc.cgi?acc=GSE4290 datasets also had no significant difference.

According to their expression profiles, gliomas are classified into four molecular subtypes: proneural, neural, classic and mesenchymal (Verhaak *et al.*, 2010). However, recent studies showed that the neural subtype lacked characteristic gene abnormalities, suggesting that the neural subtype was not tumour‐specific (Brennan *et al.*, 2013; Iser *et al.*, 2017). Compared to the proneural subtype, the mesenchymal subtype is related to poor clinical outcomes in patients (Arimappamagan *et al.*, 2013).

Therefore, based on the molecular subtype, we examined BACE2 mRNA expression. Compared to the proneural subtypes, the expression level of BACE2 in mesenchymal subtypes is significantly higher in the TCGA and CGGA databases (Fig. 1B). Additionally, the GSEA showed that mesenchyme‐related genes were significantly enriched in samples with high BACE2 expression (Fig. S2A,B). Furthermore, a ROC curve was generated to reveal the sensitivity of BACE2 protein in differentiating patients with the mesenchymal subtype from those with the nonmesenchymal subtype in the TCGA database (Fig. S1B). The optimal BACE2 expression level was 16.409 (Log_2_FPKM_UQ), and the corresponding specificity and sensitivity were 0.691 and 0.723, respectively. In addition, the levels of BACE2 mRNA and protein in the human glioma samples and normal brain tissues from Qilu Hospital (Jinan, China) were analysed by RT‐qPCR and IHC. The results showed that compared with LGG and normal brain tissues, BACE2 mRNA expression levels in GBM tissues were significantly higher (Fig. 1C). Moreover, our results indicated that the BACE2 protein levels were also increased in the GBM tissues compared with the LGGs and normal brain tissues (Fig. 1D,E). Thus, the expression level of BACE2 is associated with a higher grade of human glioma.

Furthermore, the BACE2 expression level was also related to the clinicopathological characteristics of glioma patients in the TCGA database. A high BACE2 expression level (greater than median value) was significantly associated with patient age and KPS (Table 1). As stated in several reports, the isocitrate dehydrogenase 1/2 (IDH1/2) mutation, methylation of *O*‐methylguanine‐DNA methyltransferase (MGMT) promoter methylation, codeletion of 1p/19q, telomerase reverse transcriptase (TERT) loss and alpha‐thalassaemia/mental retardation syndrome X‐linked (ATRX) mutation were associated with a better prognosis for glioma patients (Jiang *et al.*, 2016; Yan *et al.*, 2009). Therefore, we analysed whether the expression of BACE2 is associated with these molecular genetic features. High BACE2 expression was related to wild‐type IDH1 in glioma patients, while low BACE2 expression was associated with other features, including MGMT promoter methylation, 1p/19q codeletion, TERT loss and ATRX mutation. Thus, based on the above results, we suggest that high BACE2 expression indicates a worse prognosis for glioma patients.

**Table 1:** Correlation of BACE2 expression in human glioma patients with different clinicopathological features. *P* values were determined by chi‐square and Fisher's exact tests.

Variable		High BACE2 expression	Low BACE2 expression	Chi‐square values	*P* value
Age	≧ 45	215	112	54.19	< 0.001
	< 45	102	182		
Gender	Male	192	165	1.241	0.265
	Female	125	129		
KPS	≧ 80	150	160	7.779	0.005
	< 80	45	22		
WHO grade	II	64	152	137.892	< 0.001
	III	112	127		
	IV	141	15		
TCGA subtype	Proneural	59	180	178.452	< 0.001
	Classical	75	11		
	Mesenchymal	92	8		
IDH status	Mutant	123	304	210.022	< 0.001
	Wild‐type	206	29		
MGMT promoter	Methylated	178	297	81.309	< 0.001
	Unmethylated	129	36		
1p/19q	Codeletion	40	129	61.769	< 0.001
	Non‐codeletion	291	205		
TERT expression	Not expressed	122	190	24.397	< 0.001
	Expressed	202	144		
ATRX status	Mutant	68	128	23.655	< 0.001
	Wild‐type	255	205		

### 3.2 BACE2 expression correlated with poor clinical outcomes in glioma patients

We found that the LGG and GBM patients with high BACE2 expression had a worse prognosis than patients with low BACE2 expression in the TCGA database (Fig. 1F). These findings were verified with the CGGA and http://www.ncbi.nlm.nih.gov/geo/query/acc.cgi?acc=GSE16011 datasets (Fig. S1C).

In addition, univariate analysis of the Cox model showed that age, WHO grade, IDH status and BACE2 expression had significant effects on prognosis (Table 2). Multivariate analysis of the Cox model was performed with the mentioned variables. The results showed that BACE2 was an independent factor, with OS (HR = 1.591, 95% CI = 1.006–2.516, *P* < 0.05). In summary, these results strongly suggested that BACE2 might serve as a prognostic biomarker in glioma.

**Table 2:** Correlation univariate Cox regression and multivariate Cox regression of BACE2 expression for overall survival of glioma patients.

Variable	Univariate Cox regression		Multivariate Cox regression	
	HR (95% CI)	*P*	HR (95% CI)	*P*
Age				
≥ 45 vs < 45	2.737 (1.829–4.090)	< 0.001	2.463 (1.606–3.776)	< 0.001
Gender				
Female vs male	0.896 (0.602–1.176)	0.186		
WHO grade				
High vs low	8.645 (6.443–11.600)	< 0.001	3.118 (1.477–6.585)	< 0.01
BACE2 expression				
High vs low	3.839 (2.859–5.116)	< 0.001	1.591 (1.006–2.516)	< 0.05
IDH1 status				
Mutant vs wild‐type	0.108 (0.602–1.176)	< 0.001	0.242 (0.151–0.387)	< 0.001

### 3.3 Gene enrichment analysis of BACE2

**Figure 2:**
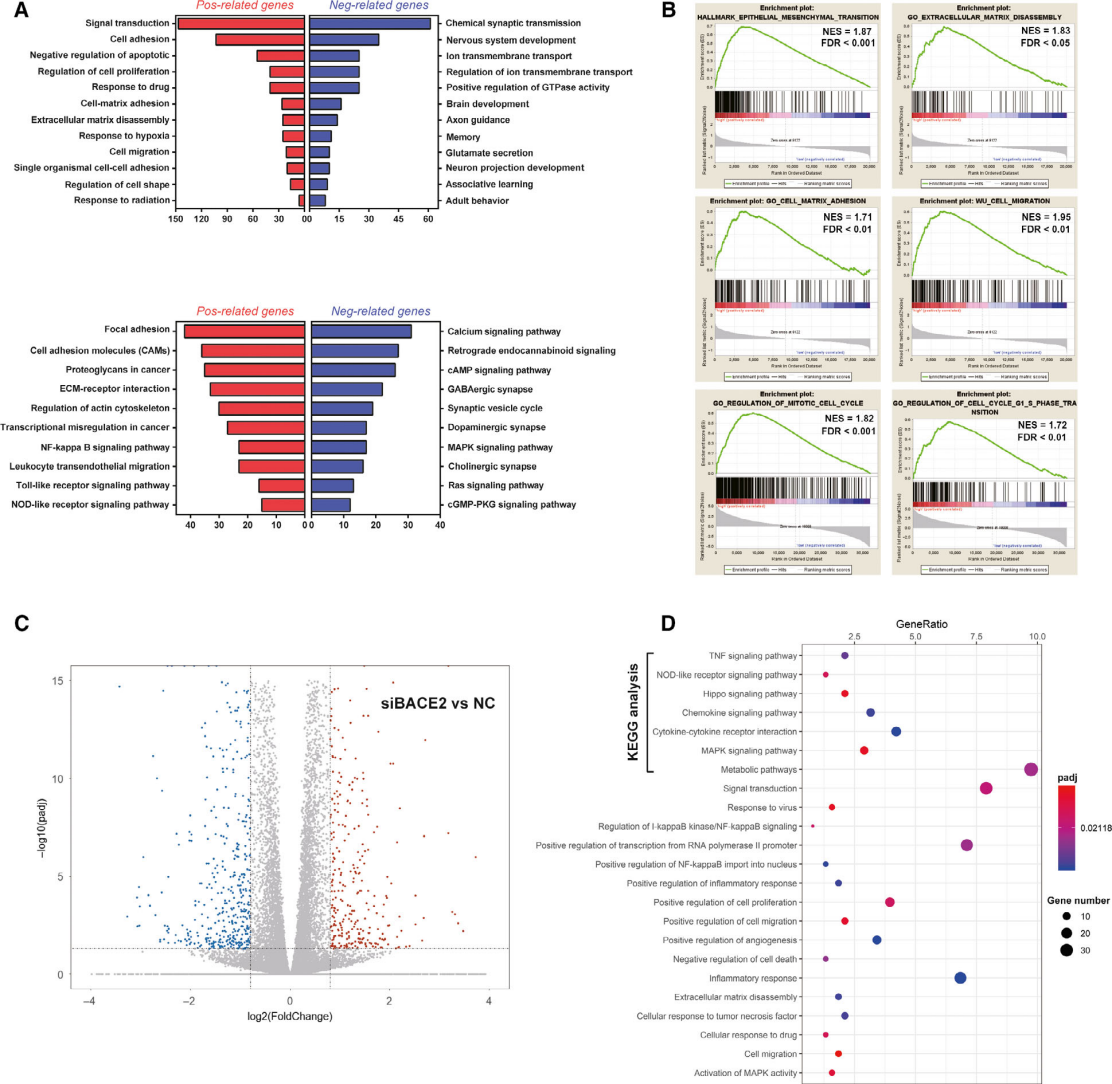
Gene enrichment analysis of BACE2. (A) A total of 1274 genes positively related to BACE2 and 694 genes negatively related to BACE2 in the TCGA database based on the correlation analysis. The GO and KEGG analyses were performed using positively and negatively related genes as shown. (B) Results of the GSEA showing that high BACE2 expression enhances EMT, tumour invasion, tumour metastasis and regulation of the cell cycle at the G1‐S phase transition. NES = normalized enrichment score, FDR = false discovery rate. (C) Distribution of peaks (fold change > 0.8 or < −0.8, *P* < 0.05) with a significant change in the mRNA expression level in BACE2‐knockdown U87MG cells compared with U87MG cells. (D) The KGEE and GO analyses were performed using downregulated genes in U87MG cells.

A correlation analysis of the whole‐genome profile of BACE2 was performed with the TCGA database to explore potential biological effects of gliomas. In the database, 1247 and 649 genes were positively and negatively related to BACE2, respectively (Table S2). Then, these related genes were subjected to enrichment analysis. Gene Ontology (GO) analysis revealed that the genes that were positively related to BACE2 were mainly involved in cancer‐promoting processes, including cell adhesion, cell proliferation, cell migration and extracellular matrix disassembly. On the other hand, the genes that were negatively associated with BACE2 were greatly enriched in cell differentiation or transport processes, such as those observed during nervous system development, axon guidance and regulation of transmembrane ion transport (Fig. 2A). The KEGG analysis indicated that BACE2 was mainly enriched in processes associated with focal adhesion, cancer‐related pathways and regulation of the actin cytoskeleton. Finally, GSEA revealed that high BACE2 expression was statistically associated with epithelial–mesenchymal transition (EMT), tumour invasion, tumour metastasis and the regulation of the G1‐S phase transition in the cell cycle (Fig. 2B).

Furthermore, we performed RNA‐seq in U87MG cells and BACE2‐knockdown U87MG cells. A total of 451 genes were specifically downregulated, while 623 were specifically upregulated in BACE2‐knockdown U87MG cells compared to U87MG cells (Fig. 2C). GO and KEGG enrichment analysis revealed that the downregulated genes were enriched mostly in the cell adhesion, cell cycle and cancer‐related pathways, which was consistent with the enrichment results in the TCGA database (Fig. 2D). In summary, these results indicated that BACE2 advanced the malignancy of glioma through these malignant processes.

### 3.4 BACE2 promotes invasion, migration and mesenchymal features in glioma cell lines

**Figure 3:**
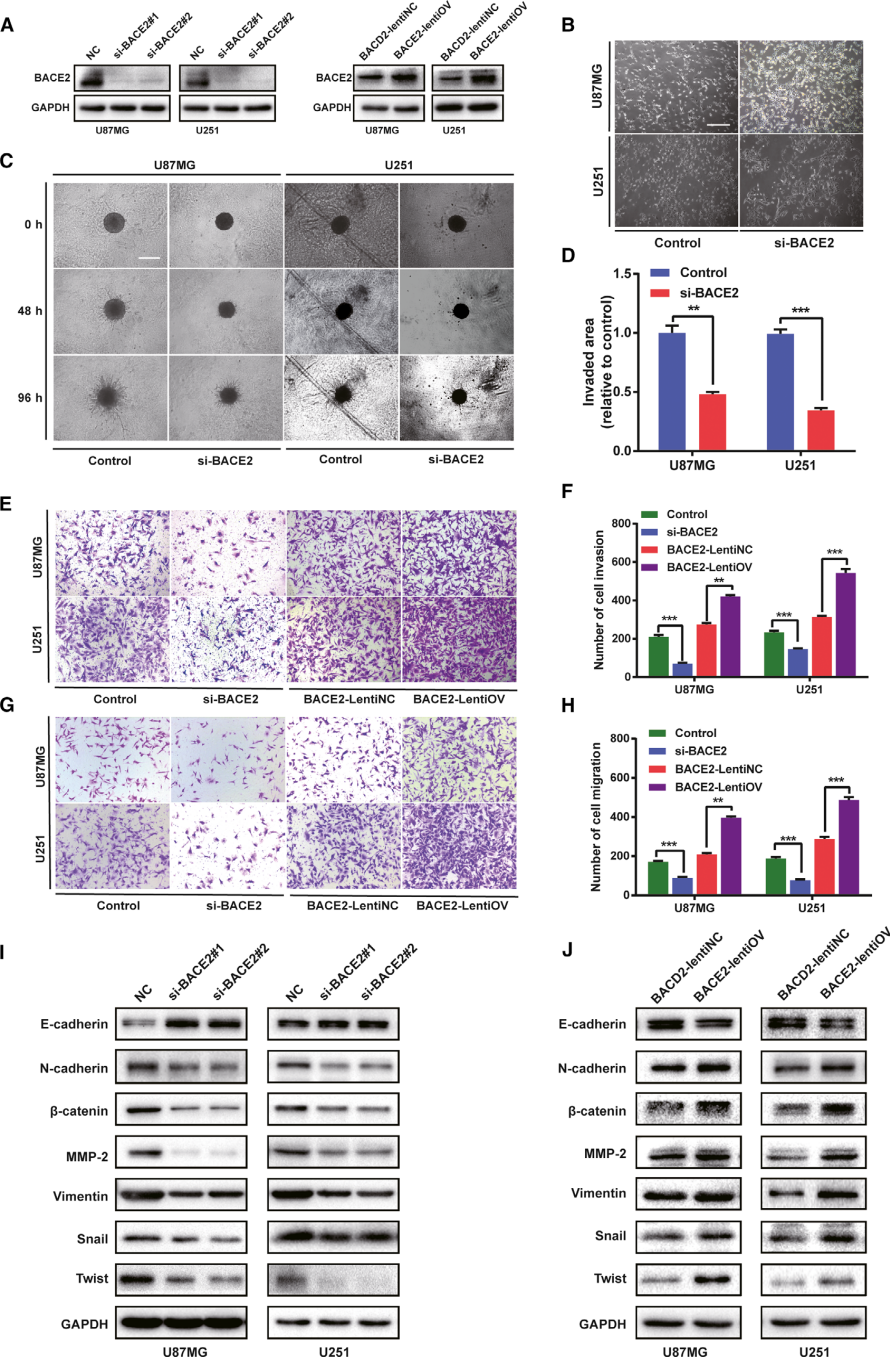
BACE2 promotes the EMT of glioma cells. (A) The expression levels of BACE2 in U87MG and U251 cells transfected with BACE2 and the siRNA control as determined by western blot analysis. The expression levels of BACE2‐LentiOV and BACE2‐LentiNC in U87MG and U251 cells were determined by western blot analysis. GAPDH was used as a loading control. (B) Representative images of U87MG and U251 cells treated with BACE2 and the siRNA control at 48 h are shown. Scale bar = 30 µm. (C) The representative images of the U87MG and U251 cell spheroids treated with BACE2 and the siRNA control evaluated at 48 h and 96 h are shown. Scale bar = 200 mm. (D) The results of the quantification of the invaded area at 96 h. (E, F) Representative images and statistical results in invasion assays of transfected U87MG and U251 cells. (G, H) Representative images and statistical results of the migration assays of the transfected U87MG and U251 cells. (I, J) The expression levels of the EMT protein marker and the related transcription factors in the transfected U87MG and U251 cells. GAPDH was used as a loading control. The data are shown as the mean ± SEM from three independent experiments. ***P < 0.001; **P < 0.01; ns, not significant.

On the basis of the previous enrichment analysis, a 3D collagen spheroid invasion assay was first performed to evaluate the role of BACE2 in cell invasion. U87MG and U251 cells were transfected with two different siRNA sequences. In addition, the BACE2‐overexpression (BACE2‐LentiOV) and control (BACE2‐LentiNC) glioma cell lines were obtained by lentivirus transfection of the U87MG and U251 cells. The transfection efficiency was examined by RT‐qPCR and western blotting (Fig. 3A and Fig. S3A). Compared to that in the control group, the invaded area was significantly reduced by knocking down BACE2 with single siRNA in the U87MG and U251 spheroids (Fig. 3C,D), while the invasion capacity was increased in the BACE2‐LentiOV groups compared with the control groups (Fig. S3B,C). Furthermore, BACE2 silencing also weakened the invasion and migration capacity of the cells in the Transwell and wound‐healing assays, whereas the capacity for invasion or migration was enhanced in the BACE2‐LentiOV cells relative to the BACE2‐LentiNC cells (Fig. 3E–H and Fig. S3D).

It is widely accepted that EMT plays an important role in the invasion and metastasis of different tumours (Chiu *et al.*, 2019; Pastushenko and Blanpain, 2019). Therefore, we explored whether BACE2 is involved in EMT. First, BACE2 silencing led to glioma cell morphological transformation. The mesenchymal phenotype of U87 and U251 cells was inhibited (Fig. 3B). Furthermore, BACE2 silencing caused a significant decrease in several mesenchymal subtype markers (N‐cadherin, β‐catenin, Vimentin), upstream transcription factors (Snail, Twist) and MMP2 and an increase in an epithelial marker (E‐cadherin) compared to the control cells (Fig. 3I). These results were verified in the BACE2‐LentiOV groups compared to the BACE2‐LentiNC groups (Fig. 3J). Additionally, as observed using immunofluorescence, knocking down BACE2 decreased the formation of invadopodia (Fig. S3E,F), an important structure in the invasive growth of cancer, in gliomas (Condeelis *et al.*, 2001). Thus, based on these results, BACE2 can promote the mesenchymal transition in gliomas.

### 3.5 BACE2 promotes the proliferation of glioma cells *in vitro*

**Figure 4:**
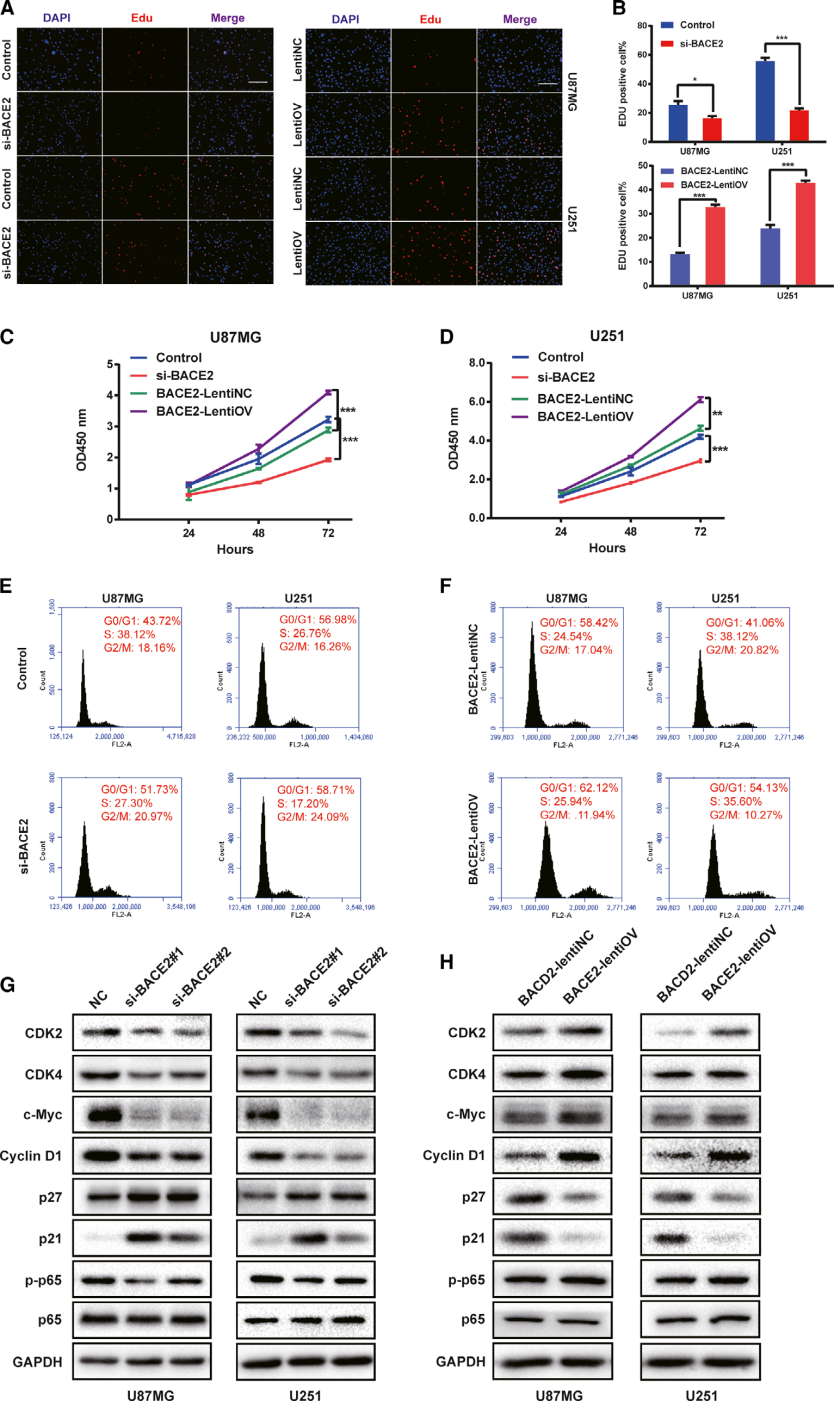
BACE2 promotes proliferation by regulating the cell cycle of glioma cells *in vitro*. (A) The results of the Edu assays performed in U87MG and U251 cells transfected with BACE2 and the siRNA control at 48 h are shown. The results of the Edu assays performed in BACE2‐LentiNC and BACE2‐LentiOV at 48 h are shown. Scale bar = 100 mm. (B) The statistical results of the Edu assay conducted with transfected U87MG and U251 cells. (C, D) Results from the CCK‐8 assay performed at 24, 48 and 96 h in transfected cells are shown with a growth curve at OD450. (E, F) The proportions of cells in different phases of the cell cycle as evaluated with PI and flow cytometry. (G, H) The expression levels of cell cycle‐related proteins and related pathway factors as determined by western blot analysis are shown. GAPDH was used as a loading control. The data are shown as the mean ± SEM from three independent experiments. ***P < 0.001; **P < 0.01; *P < 0.05; ns, not significant.

The results of the enrichment analysis indicated that BACE2 is involved in cell proliferation and the cell cycle. First, BACE2 silencing significantly decreased the percentage of positive cells compared with the control group for both the U87MG and U251 cells, and the percentage of positive cells in the BACE2‐LentiOV group was higher than that in the BACE2‐LentiNC group (Fig. 4A,B). In agreement with these findings, the results of the CCK‐8 assay showed that downregulation of BACE2 suppressed the proliferation of glioma cells, while BACE2 overexpression promoted cell proliferation (Fig. 4C,D). Furthermore, the proportion of cells in G0/G1 significantly increased in the siBACE2 group compared to that in the control group for either the U87MG or U251 cells, while BACE2 overexpression led to a decrease in the proportion of cells in G0/G1 compared to that in the control group (Fig. 4E,F). To determine the downstream target of BACE2 by western blot analysis, the expression levels of CDK2, CDK4, cyclin D1 and c‐Myc were evaluated, and the results showed that they were lower in the siBACE2 groups than in the control group and higher in the BACE2‐LentiOV group than in the BACE2‐LentiNC groups. Regarding p21 and p27 (Mitrea *et al.*, 2012), the level of cyclin‐dependent kinase inhibition was increased in the BACE2‐silenced groups and decreased in the BACE2‐overexpression group compared to the levels in their respective control groups (Fig. 4G,H). Thus, these results indicated that BACE2 promotes the proliferation of glioma cells by regulating the cell cycle.

### 3.6 BACE2 facilitates glioma progression through the NF‐κB signalling pathway

Based on the GSEA, high BACE2 expression is involved in activating the NF‐κB signalling pathway (Fig. S4A). It is well known that phosphorylation of p65 initiates activation of the canonical NF‐κB signalling pathway. Therefore, we determined the levels of p‐p65 and p65. The levels of p‐p65 and p65 were determined in transfected glioma cells, and knocking down BACE2 led to p‐p65 downregulation (Fig. 4G,H). Next, the BACE2‐LentiOV group was treated with IMD0354, an inhibitor of NF‐κB signalling. The levels of p‐p65, cyclin D1 and N‐cadherin were decreased compared to those in the groups without IMD0354 (Fig. S4B). The results from the CCK‐8 and migration assays showed that the proliferation and migration of cells in the BACE2‐LentiOV group were suppressed by the inhibition of NF‐κB signalling (Fig. S4C–E). These results indicated that BACE2 signalled through the NF‐κB signalling pathway.

### 3.7 BACE2 modulated TNF‐α‐induced NF‐κB activity through the PP1A/IKKβ axis

**Figure 5:**
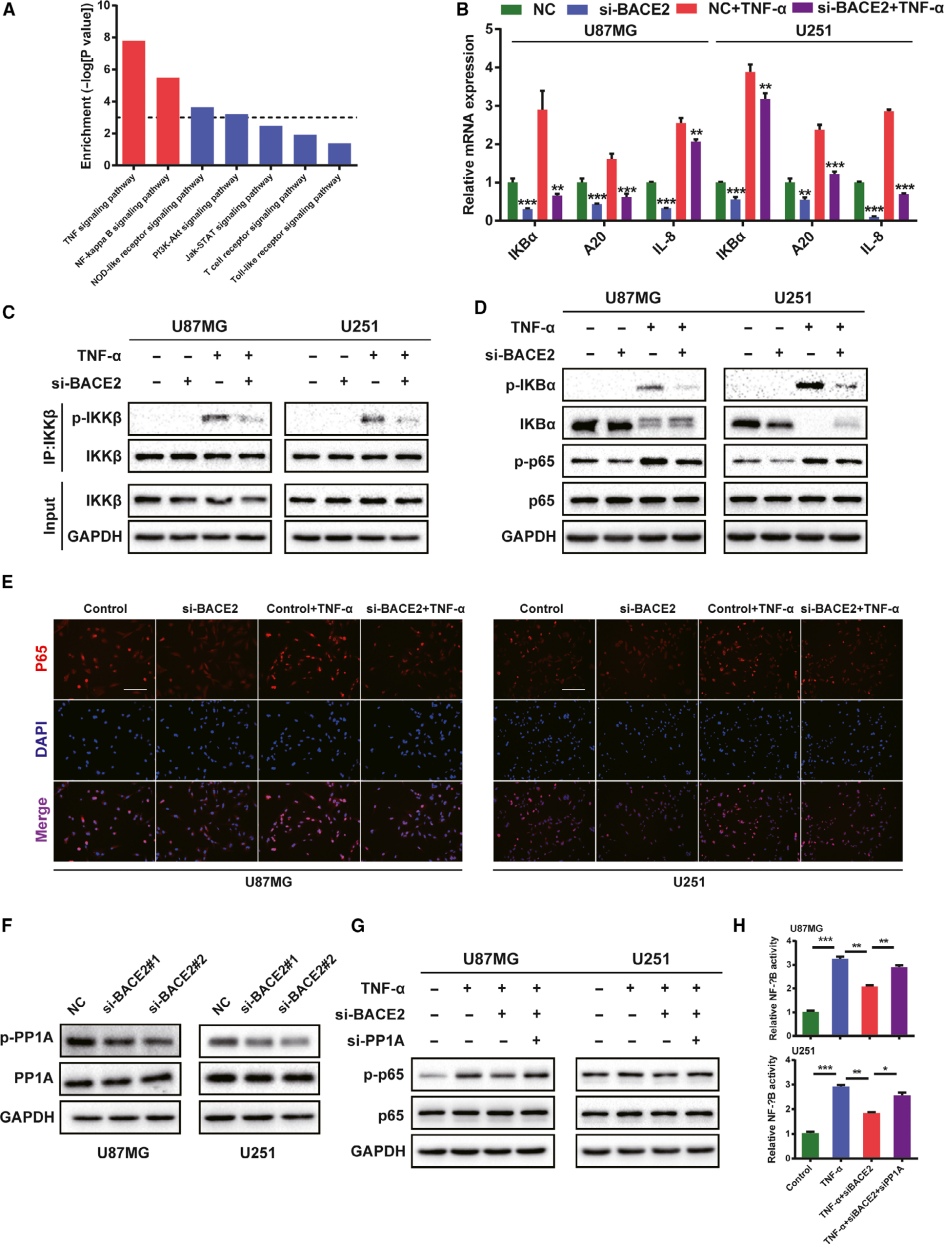
BACE2 modulates the TNF‐α‐induced NF‐κB activity through the PP1‐IKK axis. (A) The KEGG pathway analysis was performed with genes positively related and negatively related to the BACE2 genes. (B) The expression of IKBα, A20 and IL‐8 was determined by RT‐qPCR. The U87MG and U251 cells were transfected with BACE2 and the siRNA control. After 48 h, the cells were treated with or without TNF‐α (20 ng·mL^−1^) for 8 h. (C) Knocking down BACE2 prevented TNF‐α from inducing phosphorylation of IKKβ. The U87MG and U251 cells were transfected with BACE2 and the siRNA control. After 48 h, the cells were treated with or without TNF‐α (20 ng·mL^−1^) for 20 min, followed by IP. (D) The BACE2 knockdown suppressed the degradation of IKBα and the phosphorylation of p65. Glioma cells were transfected with BACE2 and the siRNA control. After 48 h, the cells were incubated with TNF‐α (20 ng·mL^−1^) for 15 min. (E) Representative images show that knocking down BACE2 prevented the NF‐κB nuclear transport that had been induced by TNF‐α. Forty‐eight hours after transfection, TNF‐α (20 ng·mL^−1^) was added, and the cells were incubated for 20 min, followed by immunofluorescence analysis. Scale bar = 100 mm. (F) Knocking down BACE2 inhibited the phosphorylation of PP1A. The glioma cells were transfected with BACE2 and the siRNA control for 48 h. (G) The PP1A knockdown prevented the downregulation of BACE2 from inhibiting TNF‐α‐induced phosphorylation of p65. The cells were treated as in Fig. 5D. GAPDH was used as a loading control. (H) Knockdown of PP1A reversed the inhibitory effect of BACE2 knockdown on TNF‐α‐induced NF‐κB activity. The cells were treated as indicated. The data are shown as the mean ± SEM from three independent experiments.***P < 0.001; **P < 0.01; *P < 0.05; ns, not significant.

Moreover, KEGG enrichment analysis indicated that BACE2 was strongly associated with TNF‐α signalling (Fig. 5A). Prior studies have noted the importance of the regulation of NF‐κB by TNF‐α through the activation of the IKK complex (Taniguchi and Karin, 2018). Thus, we investigated whether BACE2 modulated TNF‐α‐stimulated NF‐κB activity. Initially, knocking down BACE2 decreased the expression of NF‐κB downstream genes, including IKBα, A20 and IL‐8 (Fig. 5B). Furthermore, BACE2 knockdown suppressed the phosphorylation of IKKβ induced by TNF‐α in glioma cells (Fig. 5C). As IKBα and p65 are IKK substrates, we examined the phosphorylation of IKBα and p65 and found that knocking down BACE2 inhibited TNF‐α‐induced p‐IKBα and p‐p65 (Fig. 5D). In agreement with this finding, BACE2 knockdown suppressed TNF‐α‐stimulated import of p65 into the nucleus (Fig. 5E). However, how BACE2 modulates this TNF‐α‐induced NF‐κB activity remains unknown. PP1A inhibits TNF‐α‐stimulated NF‐κB activity by dephosphorylating p‐IKKβ (Li *et al.*, 2008; Qu *et al.*, 2015). Based on the TCGA and CGGA databases, we found that the expression of BACE2 was positively correlated with the expression of p‐PP1A (Fig. S4F). After BACE2 was knocked down, the expression of p‐PP1A decreased in both the U87MG and U251 cell lines (Fig. 5F). To investigate whether PP1A is involved in the regulation of BACE2 in TNF‐α‐induced NF‐κB, we determined the p‐p65 levels by western blot analysis and performed an NF‐κB reporter assay. We found that PP1A knockdown reversed BACE2 silencing and prevented TNF‐α from inducing NF‐κB activation through the PP1A/IKK pathway (Fig. 5G,H). These results showed that BACE2 regulates TNF‐α‐induced NF‐κB activity through the PP1A/IKKβ pathway.

### 3.8 TGFβ1 induces BACE2 in glioma cells

**Figure 6:**
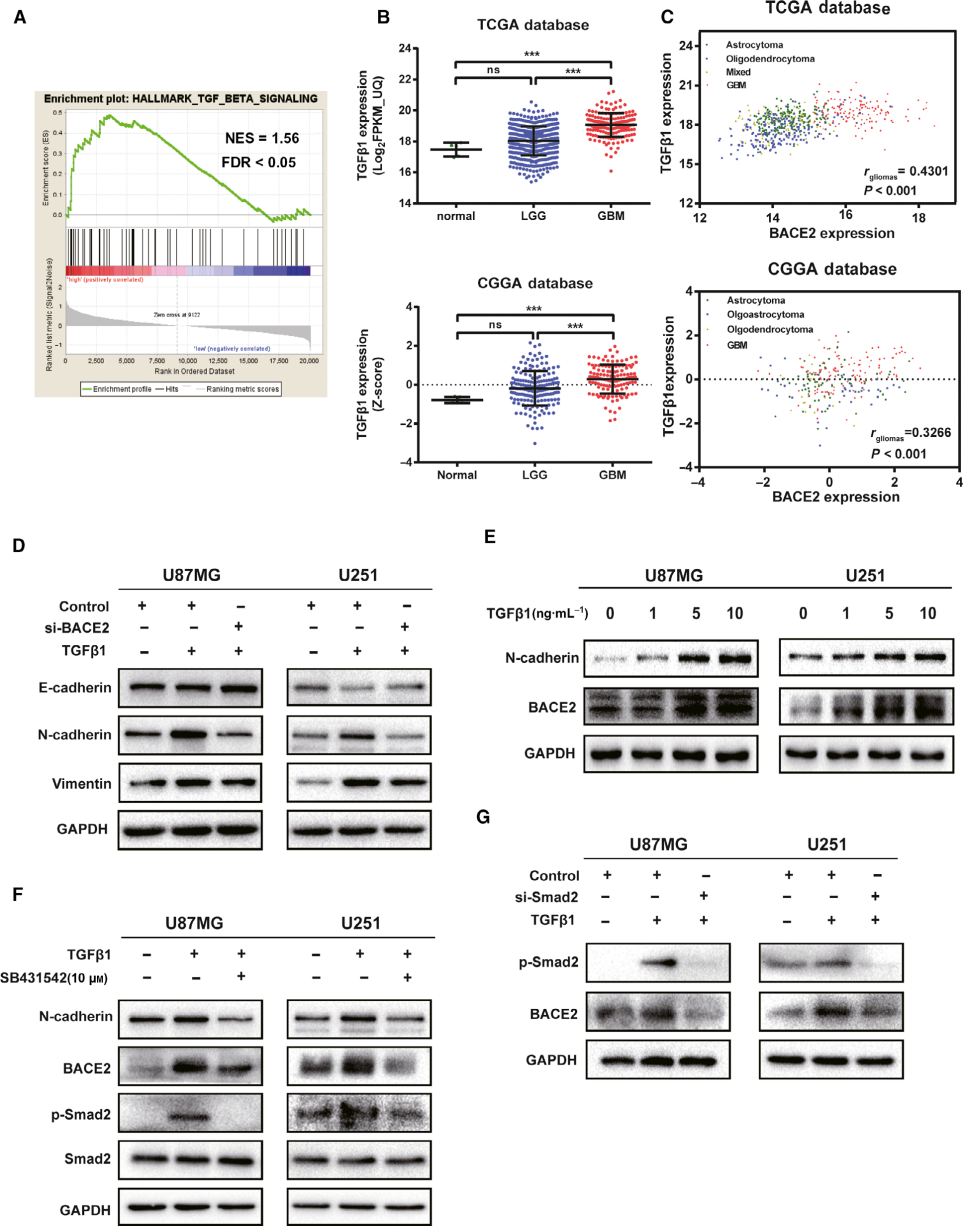
TGFβ1 promotes BACE2 expression in gliomas. (A) High BACE2 expression enhanced in the TGFβ signalling pathway according to the GSEA. (B) Results of the quantification of TGFβ1 expression in glioma tissues with the TCGA and CGGA databases. (C) The correlation between BACE2 expression and TGFβ1 expression in glioma patients according to the TCGA and CGGA database. (D) The western blots for the EMT marker in the U87MG and U251 cells transfected with BACE2 and the siRNA control in the presence of TGFβ1 (10 ng·mL^−1^) are shown. € The BACE2 expression levels with different concentrations of TGFβ1 (0, 1, 5 and 10 ng·mL^−1^) as evaluated by western blot analysis for the U87MG and U251 cells are shown. (F) The protein levels of N‐cadherin, BACE2, Smad2 and p‐Smad2 in the U87MG and U251 cells treated with TGFβ1 with or without SB431542 (10 μm) are shown as determined by western blot analysis. (G) The western blots for BACE2 and p‐Smad2 from the U87MG and U251 cells transfected with si‐Smad2 or si‐NC are shown. The results are representative of three independent experiments. ***P < 0.001; ns, not significant.

The signalling pathway that regulates BACE2 expression in gliomas is vital in exploring therapeutic treatments. Based on the GSEA, BACE2 was enriched in the TGFβ signalling pathway (Fig. 6A). TGFβ1 can regulate brain tumour progression (Bruna *et al.*, 2007; Guo *et al.*, 2017; Jennings *et al.*, 1991). Indeed, the expression of TGFβ1, as well as that of BACE2, was higher in the GBM cells than in the LGG cells in the TCGA and CGGA databases (Fig. 6B). Moreover, there was a correlation between BACE2 and TGFβ1 expression in glioma patients (Fig. 6C). Through western blot analysis, we observed that knocking down BACE2 suppressed EMT stimulated by TGFβ1 (10 ng·mL^−1^) in both U87MG and U251 cells, which indicated that BACE2 may be stimulated by TGFβ1 in glioma cells (Fig. 6D). Next, the expression of BACE2 was upregulated with increasing concentrations of TGFβ1 in the medium for both glioma cell lines (Fig. 6E). In contrast, the promoting effect of TGFβ1 on BACE2 was blocked by SB431542, a specific inhibitor of the TGFβ/Smad pathway (Fig. 6F). Furthermore, to determine whether BACE2 was regulated by TGFβ1 through the Smad pathway, the cells were transfected with si‐Smad2. In the presence of TGFβ1, the protein levels of BACE2 were decreased in the Smad2‐silenced group *in vitro* compared with the control group (Fig. 6G). Thus, the above results indicated that TGFβ1 induced BACE2 *via* the TGFβ/Smad pathway in glioma.

### 3.9 Knocking down BACE2 inhibits tumorigenesis in xenograft mice

**Figure 7:**
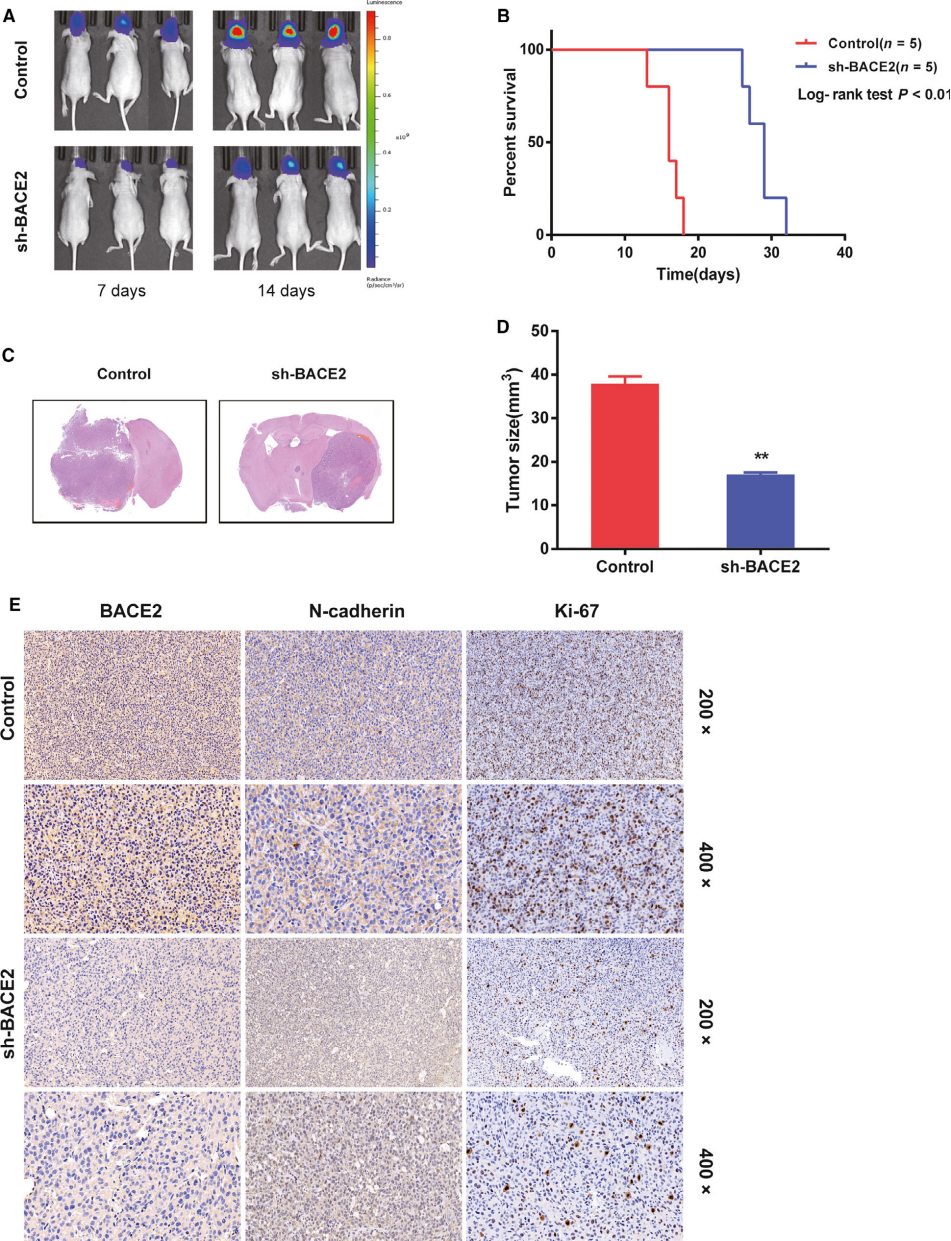
Knocking down BACE2 inhibits tumorigenesis in xenograft mice. (A) Representative bioluminescence images of the intracranial xenograft mice 7 and 14 days after implantation with U87MG cells transfected with sh‐BACE2 or the control. (B) Results from the survival analysis for mice implanted with U87MG cells transfected with sh‐BACE2 or the control. (C) Sections of mouse brains subjected to H&E staining at ~ 4 weeks after implantation of the control or sh‐BACE2 xenograft. (D) The tumour size (mm^3^) was measured. (E) The protein levels of BACE2, N‐cadherin and Ki‐67 in sections from mouse brains as determined with IHC. Magnification: 200×, upper; 400× lower. The data are presented as the mean ± SD. **P < 0.01.

To further determine the function of BACE2, we extended our investigation to experiments *in vivo*. The sh‐BACE2 cells and corresponding control cells were obtained by lentivirus transfection with luciferase‐labelled U87MG.sh‐BACE2, and they were transplanted into the brains of nude mice. The mice were imaged by *in vivo* bioluminescence 7 and 14 days after implantation (Fig. 7A). The average radiance of the tumours from the sh‐BACE2 group was significantly lower than that of the control group. The overall survival was also higher in the sh‐BACE2 group than in the control group (Fig. 7B). Similarly, the tumour size of the group with transplanted sh‐BACE2 cells was significantly smaller than that of the control group (Fig. 7C,D). The protein levels of N‐cadherin, Ki‐67 and BACE2 were lower in the sh‐BACE2 group (Fig. 7E). Thus, these results proved that the stable downregulation of BACE2 suppressed the growth and invasion of glioma in the xenograft mice.

## 4 Discussion

In this study, we investigated the function of BACE2, which is expressed at an increased level in GBM tissues compared with LGG or normal brain tissues. In addition, the expression of BACE2 was significantly upregulated in the mesenchymal molecular subtype of human glioma. Furthermore, patients with higher BACE2 expression had a poorer prognosis. In contrast, lower BACE2 expression was associated with active prognostic markers, including IDH mutation, MGMT promoter methylation, 1p/19q codeletion, TERT loss and ATRX mutation. Additionally, univariate and multivariate analysis showed that BACE2 might be an independent prognostic element in glioma. Finally, the role of BACE2 in promoting the EMT and proliferation of glioma was demonstrated through functional studies with knockdown and overexpression of BACE2.

Several reports have shown that the EMT plays a significant role in driving the invasion of tumour cells in malignant gliomas (Iser *et al.*, 2017; Liu *et al.*, 2018). The biological processes assessed and the GSEA indicated that BACE2 may, indeed, be involved in cell invasion, cell migration and the mesenchymal transition in gliomas. In this study, we observed that BACE2 could enhance invasion and migration cells in gliomas. Furthermore, mesenchymal markers, including N‐cadherin, β‐catenin and Vimentin; the epithelial marker E‐cadherin; and upstream transcription factors, including Snail and Twist, were regulated by BACE2 in gliomas. In addition, BACE2 also downregulated MMP2, which plays a significant role in type IV collagen degradation during tumour invasion and migration (Tester *et al.*, 2000). By immunofluorescence, we found that knocking down BACE2 led to F‐actin cytoskeletal changes in gliomas. This finding suggested that knocking down BACE2 reduced the formation of invadopodia by suppressing the F‐actin‐rich edge in glioma cells. Altogether, these findings suggested that BACE2 is a crucial molecule that promotes cell invasion, migration and the mesenchymal transition in human gliomas.

Cell proliferation and abnormal growth are hallmarks of human gliomas. Changes in intracellular genetic material cause cell cycle‐related proteins to become unregulated, leading to uncontrolled cell proliferation (Hanahan and Weinberg, 2011). Notably, according to the GO analysis and GSEA, BACE2 might indeed promote tumour cell proliferation through regulation of the cell cycle. The results of the analyses were confirmed in experiments *in vivo* and *in vitro*. Silencing of BACE2 in glioma cells led to cell cycle arrest at G0‐G1 and suppressed tumour growth in xenograft mice, while BACE2 overexpression decreased the proportion of cells in the G0‐G1 phase of the cell cycle. This observational study suggests that BACE2 can be used as a potential gene target for therapy in glioma patients. In addition, we investigated the potential biological process through which BACE2 promotes glioma malignant development and evaluated the expression levels of some key regulators of the cell cycle, including CDK2, CDK4, cyclin D1, p21 and p27. We found that BACE2 knockdown led to significantly decreased levels of p‐p65 and key tumour promoters, namely CDK2, CDK4, cyclin D1 and c‐Myc. In addition, inhibition of cyclin‐dependent kinase p21 and p27 expression was increased after BACE2 depletion.

Very little was found in the literature on the relationship between BACE2 expression and tumours. For instance, BACE2 is highly expressed in breast and colon tumours compared with normal tissues (Kondoh *et al.*, 2003). In addition, BACE2 expression is also increased in colorectal adenomas (Tsuji *et al.*, 2004). These studies support our findings. However, the function and potential mechanism of BACE2 require investigation in these tumours.

As observed in the literature review, NF‐κB is implicated in many hallmarks of cancer development, including growth factor‐independent proliferation, tumour invasion and metastasis and inhibition of apoptosis (Nogueira *et al.*, 2011). The GSEA and KEGG analysis predicted that BACE2 is highly involved in NF‐κB signalling. Therefore, an NF‐κB inhibitor was used to confirm this possibility. Moreover, silencing of BACE2 prevented TNF‐α‐induced activation of NF‐κB. We found that BACE2 knockdown prevented the phosphorylation of IKKβ and IKBα as well as p65 and that the degradation of IKBα was induced by TNF‐α. It was reported that PP1 dephosphorylated TNF‐α‐induced p‐IKK through a complex with CUEDC2 (Li *et al.*, 2008) and hCINAP (Qu *et al.*, 2015). We found that BACE2 is a negative regulator of PP1A. As expected, the PP1A knockdown reversed the downregulation of BACE2 by inhibiting the TNF‐α‐induced activation of NF‐κB. These results implied that BACE2 modulated TNF‐α‐induced NF‐κB activity through the PP1A/IKKβ axis.

Several reports have shown that TGFβ is a multitasking cytokine that regulates a variety of biological processes, including stemness, metastasis, angiogenesis and cell growth (Ahmadi *et al.*, 2019; Katz *et al.*, 2016). In addition, TGFβ stimulates the oncogene activity to promote EMT in some cancer types (Shao *et al.*, 2018). According to these studies, the TGFβ1/Smad signalling pathway plays a significant role in tumour development. In our study, there was a close correlation between BACE2 and TGFβ1 expression in the TCGA and CGGA cases. In the *in vitro* experiments, TGFβ1 induced BACE2 expression in two glioma cell lines. This effect can be blocked by the specific inhibitor SB431542. Furthermore, silencing of Smad2 in the presence of TGFβ1 could also suppress the induction of BACE2 in U87MG and U251 cells. These results suggest that the TGFβ1/Smad signalling pathway is an upstream regulator of BACE2 expression in gliomas. However, further research should be undertaken to investigate the potential molecular mechanisms that coordinate BACE2 and TGFβ1 signalling in gliomas.

## 5 Conclusion

**Figure 8:**
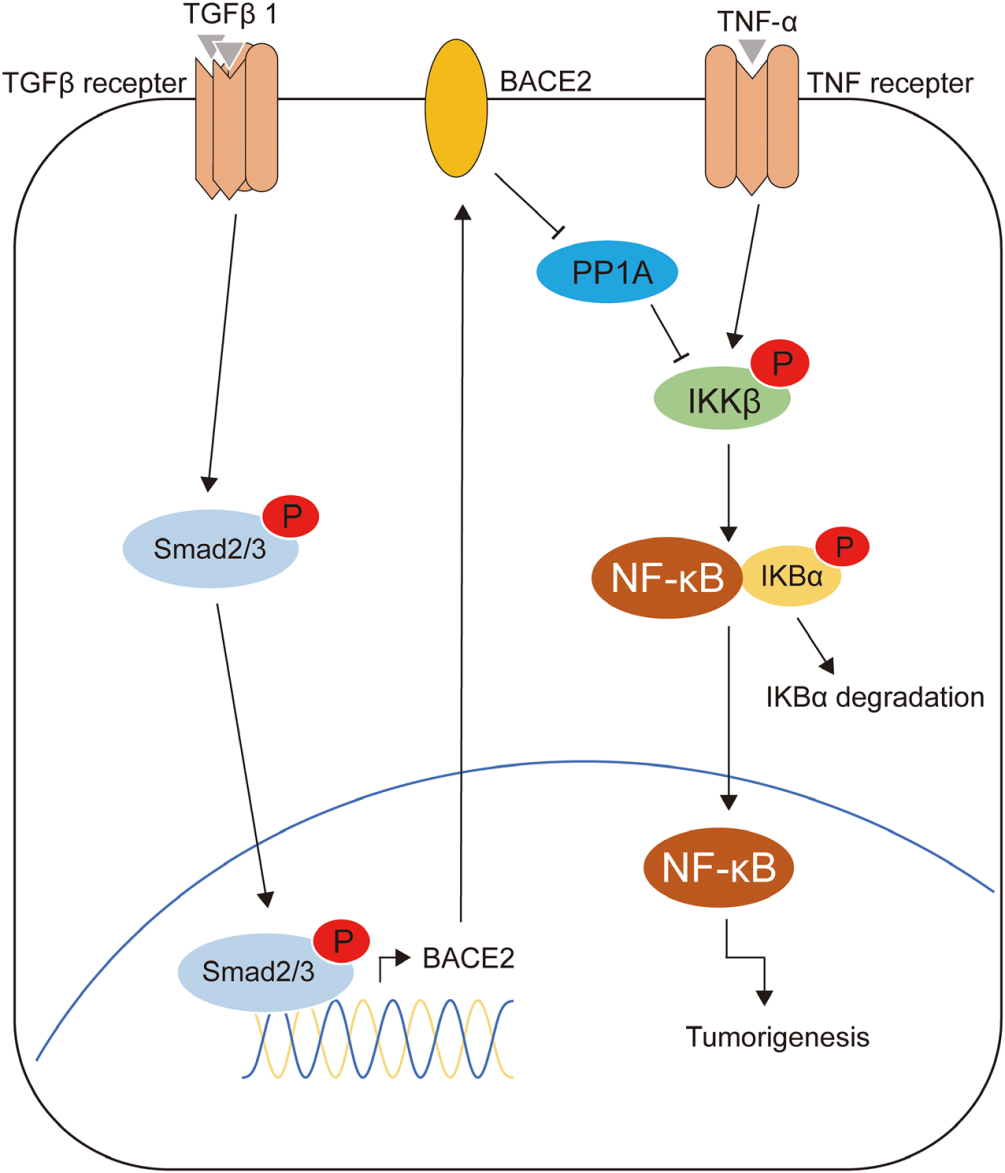
Schematic diagram of BACE2‐regulated pathway in glioma cells.

We demonstrated for the first time that higher levels of BACE2 expression are associated with a higher grade of human glioma, the mesenchymal molecular subtype of GBM and a worse prognosis. Furthermore, TGFβ1 stimulates BACE2 expression through Smad‐dependent signalling, which modulates TNF‐α‐induced NF‐κB activity through the PP1A/IKK pathway to promote tumorigenesis in glioma cells (Fig. 8). BACE2 may serve as a novel biomarker and a potential therapeutic target in the treatment of human glioma.

## Conflict of interest

The authors declare no conflict of interest.

## Author contributions

HW, GX and HX designed and performed the experiments. HW, SW, MQ, WQ and ZZ analysed the results. HW, RZ, SZ and YQ organized the results and wrote the paper. HW, RZ, HZ, XG and XS provided the tools and patient specimens and edited the manuscript. All authors participated in critical revision of the manuscript for important intellectual content.

## Supporting information


**Fig. S1.** The prognostic values of BACE2 in validated cohorts.
**Fig. S2.** High expression of BACE2 enriched in mesenchymal subtype gliomas.
**Fig. S3.** BACE2 enhanced the invasion capacity of glioma cells *in vitro*.
**Fig. S4.** Regulatory effects of BACE2 on the NF‐κB signalling pathway.

**Table S1.** The primers used in RT‐qPCR.

**Table S2.** Positively related genes and negatively related genes of BACE2 in TCGA.
