# Supplementary material for: TGFβ1‐induced beta‐site APP‐cleaving enzyme 2 upregulation promotes tumorigenesis through the NF‐κB signalling pathway in human gliomas
Source: Mol Oncol. 2020 Jan 7;14(2):407–25. doi: 10.1002/1878-0261.12623 (PMC6998390; doi:10.1002/1878-0261.12623)
Supplement: Supplementary file 1 — Fig. S1. The prognostic values of BACE2 in validated cohorts. Fig. S2. High expression of BACE2 enriched in mesenchymal subtype gliomas. Fig. S3. BACE2 enhanced the invasion capacity of glioma cells in vitro. Fig. S4. Regulatory effects of BACE2 on the NF‐κB signalling pathway. [file MOL2-14-407-s001.docx]

**Appendix A. Supplementary data**

**The following are the Supplementary data for this article:**


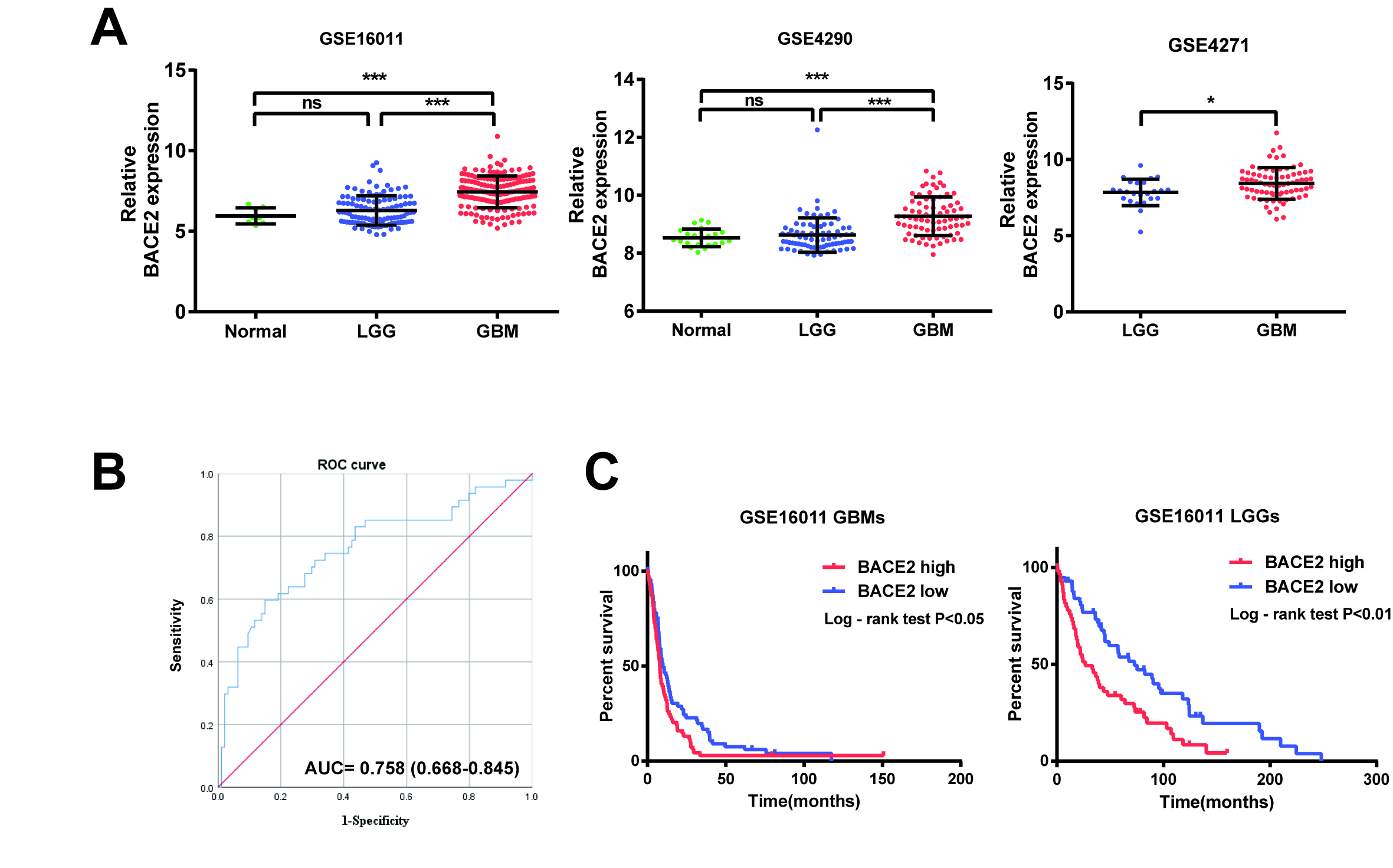


**Supplementary Fig. 1.** **The prognostic values of BACE2 in validated cohorts.**

**A** Results of the quantification of BACE2 expression in glioma tissues in GSE16011, GSE4290 and GSE4271 datasets.

**B** The ROC curve showing the sensitivity of BACE2 to differentiate mesenchymal subtypes patients from non-mesenchymal subtype patients.

**C** The Kaplan-Meier survival curves were performed with the GSE16011 dataset. The cut-off was the median BACE2 expression level. *** P < 0.001; ** P < 0.01; * P < 0.05; ns, not significant.


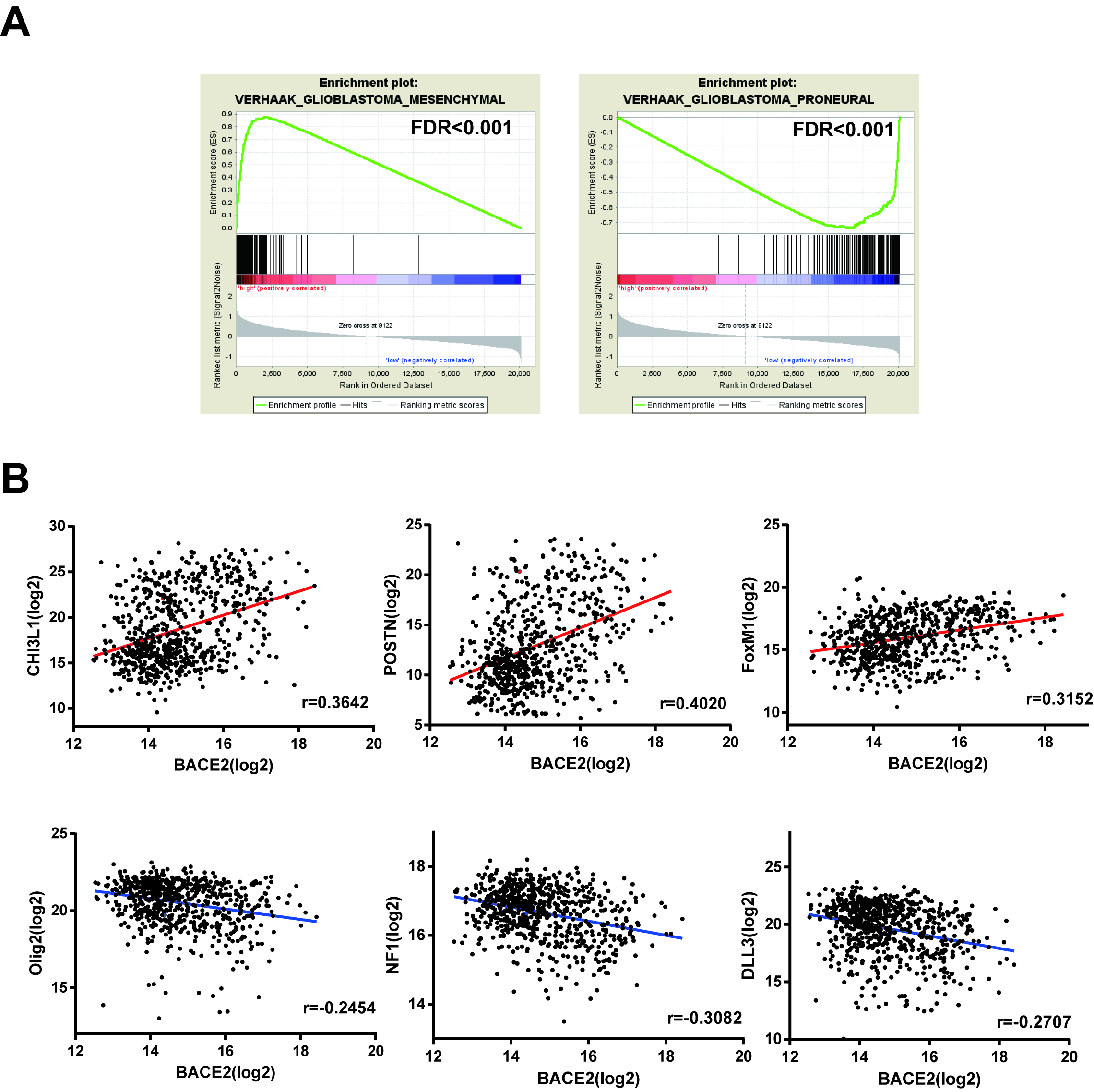


**Supplementary Fig. 2. High expression of BACE2 enriched in mesenchymal subtype gliomas. A** The GSEA analysis showed that mesenchyme-associated genes were significantly enriched with increased BACE2 expression.

**B** The BACE2 expression levels were positively correlated with mesenchymal markers (CHI3L1, POSTN, and FoxM1) but negatively correlated with pro-neural markers (Olig2, DLL3, and NF1).


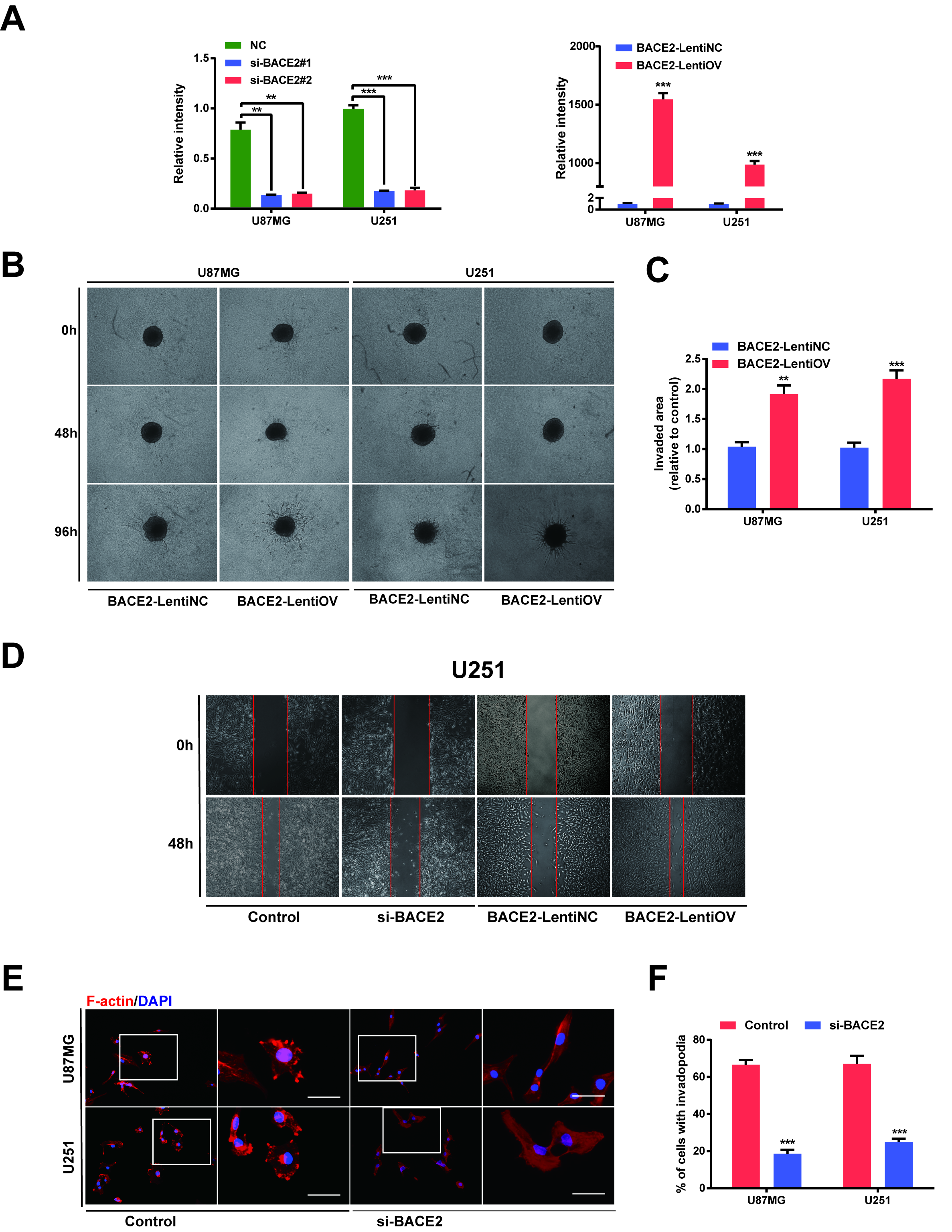


**Supplementary Fig. 3. BACE2 enhanced the invasion capacity of glioma cells in vitro.**

**A** The expression levels of BACE2 in the U87MG and U251 cells transfected with BACE2 and the siRNA control, and the expression levels of BACE2-LentiOV and BACE2-LentiNC in the U87MG and U251 cells as determined by RT-PCR are shown. GAPDH was used as a loading control.

**B** Representative images are shown of BACE2-LentiOV and BACE2-LentiNC expression levels in the U87MG and U251 cell spheroids as evaluated at 48 h and 96 h. Scale bar = 200 mm.

**C** Results from the quantification of the invaded area at 96 h.

**D** A wounding healing assay was used to investigate the migration capacity of after transfection with siRNA or lentivirus. Scale bar = 200 µm.

**E** Representative immunofluorescence images of F-actin and nuclear stain in the U87MG and U251 cells transfected with BACE2 and the siRNA control after 48 h. Scale bar = 30 µm.

**F** Invadopodium formation was quantified as a percentage of 50 randomly selected cells with invadopodia from each group. The results are representative of three independent experiments. *** P < 0.001; ** P < 0.01; *,P < 0.05; ns, not significant.


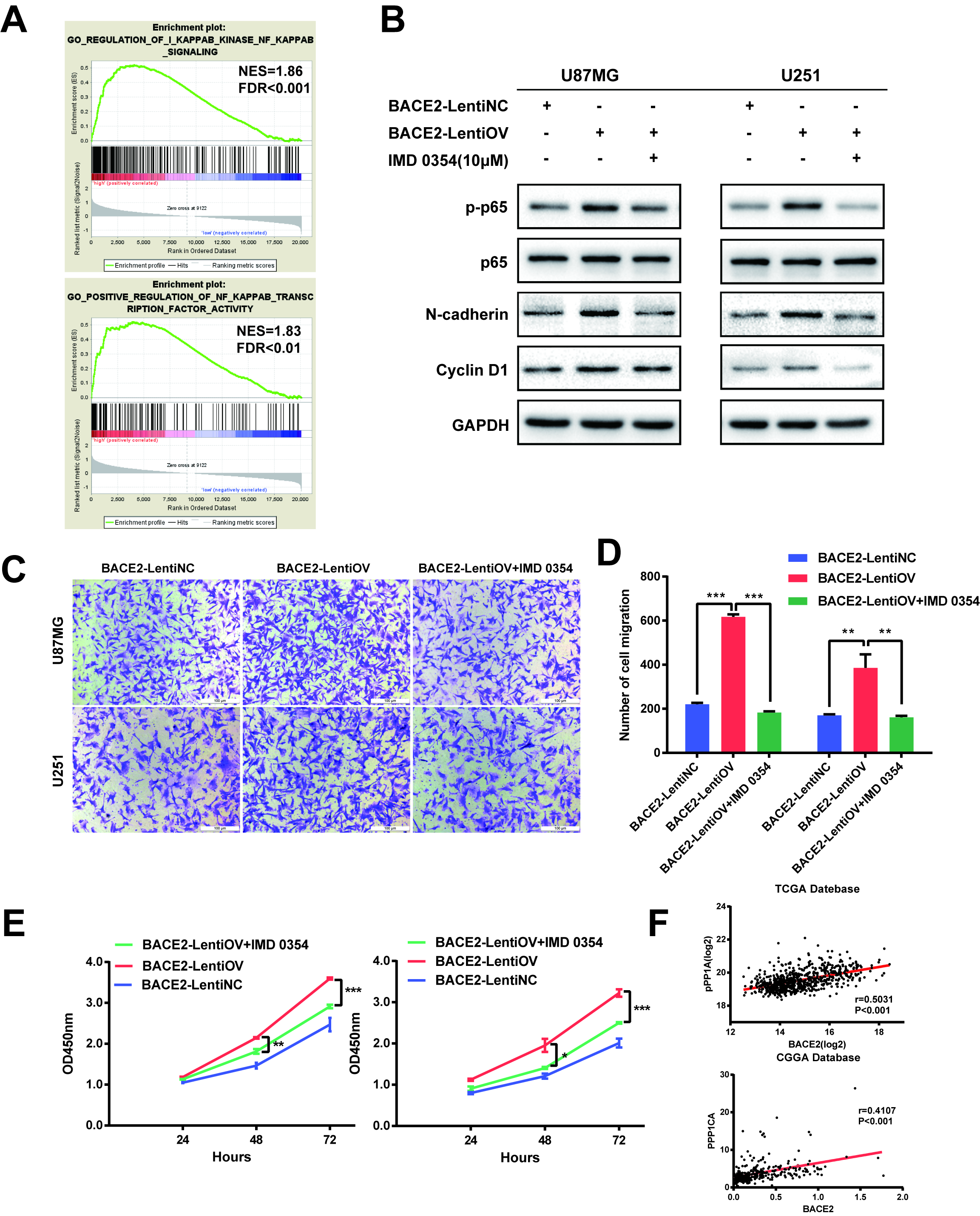


**Supplementary Fig. 4. Regulatory effects of BACE2 on the NF-κB signalling pathway.**

**A** The results of GSEA analysis showing that high BACE2 expression enhances activation of the NF-κB signalling pathway.

**B** Expression of BACE2-LentiOV and BACE2-LentiNC in the U87MG and U251 cells with or without the NF-κB inhibitor IMD0354 (soluble in DMSO to 10 μM) for 24 h. The expression levels of the downstream molecules of NF-κB signalling, Cyclin D1 and N-cadherin were determined by Western blot. GAPDH was used as a loading control.

**C** The migration capacity of glioma cells was determined with a migration assay after treatment with the NF-κB inhibitor IMD0354 for 24 h.

**D** Results from the quantitative migration assay.

**E** Cell proliferation was determined with a proliferation assay after treatment with the NF-κB inhibitor IMD0354 for 24 h.

**F** The correlation between BACE2 expression and p-PP1A expression in glioma patients according to the TCGA and CGGA database. The data are shown as the mean ± SEM from 3 independent experiments. *** P < 0.001; ** P < 0.01; * P < 0.05; ns, not significant.
