# Supplementary material for: TGFβ1‐induced beta‐site APP‐cleaving enzyme 2 upregulation promotes tumorigenesis through the NF‐κB signalling pathway in human gliomas
Source: Mol Oncol. 2020 Jan 7;14(2):407–25. doi: 10.1002/1878-0261.12623 (PMC6998390; doi:10.1002/1878-0261.12623)
Supplement: Supplementary file 2 — Table S1. The primers used in RT‐qPCR. [file MOL2-14-407-s002.docx]

**Supplementary Table S1.** The primers used in real-time qPCR.

| Gene | Sequence |
| --- | --- |
| BACE2 | 5′CGTTTTCTCCATGCAGATGAGTGT3′(F),5′CCTCCGTTGGTCCCCAGATC3′(R). |
| IKBα | 5′CCCTACACCTTGCCTGTGAG3′(F), 5′CACCAAAAGCTCCACGATGC3′(R). |
| A20 | 5′GGCCTACAACCCGCATACAA3′(F), 5′GGTCTGATCTCTCTTGGCGG3′(R). |
| IL-8 | 5′GTGTGAAGGTGCAGTTTTGCC3′(F), 5′TTTCTGTGTTGGCGCAGTGT3′(R). |
| GAPDH | 5′CCATCTTCCAGGAGCGAGATC3′(F), 5′GCCTTCTCCATGGTGGTGAA3′(R). |
